# Surgical Ventricular Restoration to Reverse Left Ventricular Remodeling

**DOI:** 10.2174/157340310790231626

**Published:** 2010-02

**Authors:** Serenella Castelvecchio, Lorenzo Menicanti, Marisa Di Donato

**Affiliations:** 1 Dept. Cardiac Surgery, IRCCS, San Donato Hospital, Milan, Italy; 2 Dept. Critical Care Medicine, University of Florence, Florence, Italy

**Keywords:** Myocardial infarction, Left ventricular remodeling, Surgical ventricular restoration, Systolic function, Diastolic function.

## Abstract

Heart failure is one of the major health care issues in the Western world. An increasing number of patients are affected, leading to a high rate of hospitalization and high costs. Even with administration of the best available medical treatment, mortality remains high. The increase in left ventricular volume after a myocardial infarction is a component of the remodeling process. Surgical Ventricular Restoration (SVR) has been introduced as an optional therapeutic strategy to reduce left ventricular volume and restore heart geometry. So far, it has been established that SVR improves cardiac function, clinical status, and survival in patients with ischemic, dilated cardiomyopathy and heart failure. Since its first description , SVR has been refined in an effort to standardize the procedure and to optimize the results. This review will discuss the rationale behind surgical reversal of LV remodeling, the SVR technique, its impact on cardiac function and survival, and future expectations.

## INTRODUCTION

Heart failure (HF) remains a major public health problem. Almost 5 million patients in the United States are affected, and 30 to 40% of patients die from HF within 1 year after receiving the diagnosis [1]. The incidence and prevalence of heart failure continues to increase due in part to an extended average life expectancy and morbidity and mortality remain high despite improvement in treatment. HF is the leading cause of hospitalization for persons over 65 years of age, and rates of hospital readmission within 6 months range from 25% to 50%, resulting in a large economic burden [2, 3]. Heart transplantation remains the treatment of choice for patients with medically refractory end stage HF [4, 5]. However, the need for immunosuppression and the paucity of donors have greatly restricted the selection criteria, leaving many patients and physicians seeking other options. Medical therapy, targeted to block the neurohormonal pathway, has dramatically improved the survival of HF patients by slowing the progression of the disease [6-8]. Despite the application of the best available medical therapy, the percentage of patients suffering from signs and symptoms of HF still remains high. This supports the concept that HF progresses independently of neurohormonal activation due to an abnormal and excessive increase in left ventricular (LV) volume. This theory, the biomechanical model of HF, was first proposed by Mann and Bristow [9]. The concept of a biomechanical model of HF introduces the need for optional strategies aimed at reducing LV volumes, and restoring heart geometry. Surgical ventricular restoration (SVR) has been introduced to restore LV shape, size, and function in patients with ischemic dilated cardiomyopathy and HF. The technique, initially introduced by Dor [10] and Jatene [11], has been refined over the last ten years in an effort to standardize the procedure and optimize the results. This review will discuss the rationale to surgically reverse LV remodeling, the SVR technique, its impact on cardiac function and survival, and future expectations.

## LV REMODELING

Left ventricular remodeling is the process by which mechanical, neurohormonal, and possibly genetic factors, alter ventricular size, shape, and function. Remodeling occurs in several clinical conditions, including myocardial infarction (MI), cardiomyopathy, hypertension, and valvular heart disease.

Myocardial infarcts (MI), particularly large, transmural infarcts, result in a number of structural changes involving both the infarcted and non-infarcted zones [12]. LV remodeling usually begins within the first few hours after an MI and may progress over time. Abnormal thinning (called “infarct expansion”) and dilatation of the necrotic zone is initially considered a compensatory mechanism to maintain stroke volume as ejection fraction declines. It is accompanied by a secondary volume-overload eccentric hypertrophy of the non-infarcted remote regions that should counteract the increased wall stress and reduce the stimulus for further dilatation [13, 14]. However, a negative balance, related to the infarct size and the degree of myocardial cells loss, towards LV enlargement may result in loading conditions that promote further dilatation and global ventricular dysfunction [12]. Structural and geometric ventricular changes proceed along with increased myocyte stress, neurohormonal activation, collagen synthesis, fibrosis, and remodeling of the extracellular matrix, resulting in further deterioration of cardiac function [15]. 

## SVR RATIONALE

SVR has been developed with the goal of improving cardiac function through a reduction in LV wall tension in accordance with the principle of Laplace’s law. Since LV wall tension is directly proportional to the LV internal radius and pressure, and inversely proportional to wall thickness, any intervention to optimize this relationship would be beneficial in terms of improving wall compliance and reducing filling pressure. Optimization may also be beneficial in terms of enhancing the contractile performance of the LV by increasing the extent and velocity of systolic fiber shortening [16].

## SVR TECHNIQUE

After the first description of the linear suture by Cooley in 1958 [17], and the circular external suture described by Jatène in 1984 [11], Dor started to use a circular patch to reconstruct the LV ("endoventricular circular patch plasty" - EVCPP) [10, 18]. This technique, completed during cardioplegia, involved opening the ventricle in the center of the depressed area and performing a thrombectomy when indicated. Then, exclusion of the dyskinetic or akinetic LV free wall proceeded with an endoventricular circular suture passed through the fibrous tissue above the transitional zone. In the event of recurrent ventricular arrhythmias, cryotherapy was applied at the transitional zone. A Dacron patch lined with pericardium was secured at the junction of the endocardial muscle and scarred tissue, thereby excluding non contractile portions of the LV and septum. The excluded scar was folded over the patch to assure hemostasis. Myocardial revascularization was performed before reconstruction, making sure to revascularize the proximal left anterior descending segment in addition to performing mitral valve repair when indicated. To avoid excessive resection, leaving too small a residual volume, Dor introduced the use of an intraventricular balloon. The balloon was filled to a volume of 60 mL/m^2^ for preoperative LVEDV < 150 mL/m^2^ or 70 mL/m^2^ for preoperative LVEDV > 150 mL/m^2^. It was removed before closure of the ventricle. Later, the procedure was adopted by many skilled and creative surgeons without real standardization, making results difficult to compare. McCarthy described a no-patch, double purse-string suture technique [19]; Mickleborough described a tailored scar excision, with septoplasty when indicated for dyskinetic septum, and modified linear closure [20]; Menicanti adopted a technique that is similar to the Dor procedure except for the use of a pre-shaped mannequin (TRISVR ^TM^, Chase Medical Richardson, TX), which is illustrated in Fig. (**1**) [21]. The mannequin is useful when the ventricle is not extremely enlarged to reduce the risk of the residual cavity being too small. It is also useful when the transitional zone between scarred and non-scarred tissue is not clearly demarcated, as in dilated cardiomyopathy and recent MI. 

The mannequin is removed before closure of the LV cavity, which is done with a direct suture if it is less than 3 cm large, or with an elliptical, synthetic patch if greater than 3 cm. Regardless, the mannequin is useful in giving the surgeon the correct position of the apex and avoiding sphericalization of the ventricle. The reconstruction of the apex can be difficult when the apical and inferior regions are severely dilated. To overcome this difficulty, Menicanti applied a modification of the Dor procedure that involves plication of the distal inferior wall before patch placement, thus placing the apex in a more anterior position as illustrated in Fig. (**2**) [21, 22].

When indicated, the mitral valve is repaired through the ventricular opening with a double arm stitch running from one trigone to the other, embedding the two arms in the posterior annulus of the mitral valve. After that, the suture is tied to undersize the mitral orifice [23]. 

## SVR, CARDIAC FUNCTION AND SURVIVAL

The first consistent results using SVR were reported by Dor and co-authors. They showed that the procedure improves LV function, NYHA functional class, and survival by reducing ventricular volumes and increasing the ejection fraction (EF). These results were observed not only in patients with classic dyskinetic aneurysms, but also in those with dilated ischemic cardiomyopathy and severe LV dysfunction. [24-26]. More interestingly, our group has demonstrated, in a large series of patients (245) treated with the Dor procedure, that surgical outcomes relate to the extent of LV asynergy and not the type of asynergy (akinetic vs. dyskinetic) (the mortality rate ranged between 12.2 % and 12.5% in large LV aneurysms, vs. 4.8% and 0% in small LV aneurysms, with an overall mortality rate of 6.8% reported in the first 562 consecutive patients of Dor experience) [27]. A few years later the first international registry, the RESTORE Group (Reconstructive Endoventricular Surgery, returning Torsion Original Radius Elliptical shape to the left ventricle), confirmed the safety and the efficacy of SVR in 1,198 patients who underwent the procedure between 1998 and 2003 [28]. Akinesia was present in 66% of the cases and up to 73.3% among patients with LVESV ≥ 80ml/m^2^. This study reported an improvement in EF from 29.6 ± 11.0% preoperatively to 39.5 ± 12.3% postoperatively (p < 0.001) and a decrease in LVESVI from 80.4 ± 51.4 ml/m^2^ preoperatively to 56.6 ± 34.3 ml/m^2^ postoperatively (p < 0.001). Thirty-day mortality after SVR was 5.3%, with this value being higher among patients in whom mitral valve repair was performed along with SVR (8.7%), versus patients in whom no mitral valve procedure was required (4.0%, p < 0.001). The overall five-year survival was 68.6 ± 2.8%. After five years, 78% of patients were not readmitted to the hospital for CHF. Surprisingly, in 2004, Mickleborough reported the results from a smaller group of patients (245) showing a lower in-hospital mortality rate (2.8%); one, five, and ten- year survivals were 92%, 82%, and 62% [20]. However, besides the fact that the technique was different from the Dor procedure, it should be pointed out that severe mitral regurgitation (MR) was considered a relative contraindication in that study; only six patients (2%) had mitral valve surgery. Excellent results have been reported by O’Neill and colleagues from the Cleveland Clinic as well [29]. The authors published data obtained from 220 consecutive patients who underwent SVR. Seventeen percent of them had an ICD implanted in situ preoperatively, 49% had associated mitral valve surgery, and 7% required an intraaortic balloon pump postoperatively. The 30-day mortality was 1% and survival at one, three, and five years was 92%, 90%, and 80%, respectively. However, the lack of the information on the type of asynergy, or on its extension, makes these results difficult to compare with those from previous series. More recently, our group reported the largest single-center study of surgical anterior restoration (1,161 patients) showing a 30-day cardiac mortality of 4.7% [30]. Patients requiring mitral valve repair/replacement (18%) had a significantly higher (13% vs. 3.0%, p < .001) operative mortality rate in agreement with the results from the RESTORE. In a subgroup of 254 patients, we reported that MR alone does not significantly increase operative mortality risk. Conversely, if associated with NYHA class III/IV, it is associated with a significant increase in the risk of mortality. If severe diastolic dysfunction is also present (E/A >2), the risk is further increased, demonstrating for the first time, that severe diastolic dysfunction may increase the risk of SVR. Table **1** shows a summary of the data from the literature.

### SVR and Mitral Regurgitation

How and when mitral regurgitation should be corrected during SVR, is still a topic of debate. In the failing heart, mitral regurgitation occurs secondarily to annular dilatation, altered left ventricular geometry, and papillary muscle dysfunction [40, 41]. Volume overload causes progressive left ventricular and annular dilatation, worsens mitral regurgitation, and decreases survival [42, 43]. It is generally accepted that moderate to severe MR (grade 3-4+) is an indication for surgical repair in conjunction with SVR. However, it has been reported that adding mitral repair to SVR with or without CABG, or to CABG alone, increases the operative risk [44-47]. Sartipy and colleagues confirmed a higher operative mortality (16%) in patients with mild-to-severe MR undergoing mitral repair in conjunction with SVR [46]. Overall survival was also significantly lower in these patients compared with patients undergoing SVR without the mitral valve procedure. Moreover, in the series by Sartipy, the MR was mild (2+) in the majority of patients (18/31).

SVR has the potential to improve mitral functioning by reducing LV volumes, papillary muscles distances (which is a main determinant of functional MR), and rebuilding a more normal heart geometry [21, 48, 49]. Recently, our group addressed the effectiveness of SVR on unrepaired mild ischemic mitral regurgitation. Our paper showed that SVR improves mitral functioning by improving geometry abnormalities [50]. Overall mid-term survival, including early mortality, was 93% at 1 year and 88% at 3 years. This is higher than would be expected in patients with post-infarction dilated ventricles and depressed left ventricular function; suggesting that mitral repair in conjunction with SVR may be unnecessary in such patients. A larger population and longer follow-up are needed to make the results conclusive. 

### SVR and Diastolic Function

Despite excellent contributions supporting SVR as a therapeutic strategy for patients with ischemic HF, few data are available on LV diastolic function (DF) in patient undergoing SVR. The ventricular remodeling following an acute myocardial infarction is accompanied by changes in diastolic properties of the LV due to scar formation. This, in turn, increases chamber stiffness and compensatory hypertrophy of the remote zone, which causes delayed relaxation [51]. In turn, the resulting increased filling pressure within the ventricle may be responsible for LV dilatation. Experimental studies have suggested surgical volume reduction has an adverse effect on DF [52, 53]. Tulner and colleagues reported data obtained from pressure-volume loop analysis before and after SVR suggesting an improvement in systolic function and changes in diastolic properties as evidenced by an increased stiffness constant [54]. However, the study was conducted under cardioplegia that could be partially responsible for interstitial edema and the observed increase in diastolic chamber stiffness. This was also suggested by Ratcliffe and Guy [55]. Previously, our group reported data obtained from invasive simultaneous pressure-volume measurements on thirty consecutive patients before and approximately 10 days after surgery [56]. We showed an improvement in diastolic function after SVR demonstrated by a significant increase in the peak filling rate (PFR) and a decrease in the constant of pressure decay (Tau). This discrepancy with the study of Tulner may be due to different time intervals of the postoperative invasive evaluation or to the patient profile. In Tulner’s study group, the patients were older and 100% were NYHA class III-IV with an EF <30%. In contrast, in our study group, the patients were younger and 43.3% of them were in NYHA class I-II. Most importantly, 63.3% had a dyskinetic scar which is a more compliant tissue and its resection or exclu-sion does not affect DF that may eventually improve [57]. 

It is reasonable to assume that the geometric implications, as well as the volume of the residual LV cavity, may affect the diastolic function in patients undergoing SVR. We addressed this issue in a recent paper [58]. One hundred and forty-six patients received a complete echocardiographic examination before and after SVR, and at the time of discharge (7 to 10 days after surgery). Diastolic function was explored using the transmitral flow velocity pattern, and four classes were defined: normal, abnormal relaxation, pseudonormal, and restrictive pattern. Diastolic function was defined as unchanged (no difference in diastolic pattern), improved (at least one class less) or worsened (at least one class more or, in the case of preoperative restrictive pattern, an E/A ratio increase of at least 20%). After SVR, the filling pattern was unchanged in 105 cases (72%), improved in 14 (9.6%), and worsened in 27 (18.4%). Based on univariate analysis, the preoperative conicity index (CI, as obtained from the apical to short axis ratio in the 4-chamber view, Fig. (**3**) [59] and the end-diastolic volume difference (the result of surgical volume reduction) were associated with a worse diastolic pattern. This means that globally dilated LV cavities (CI < 1) are more likely to worsen the diastolic function compared to LV cavities equally dilated but mostly at the apical level (CI > 1) due to the presence of a dyskinetic scar. 

### SVR and LV Dyssynchrony

The complex sequence of the events leading to LV remodeling involves the presence of electrically unexcitable scars. This predisposes the LV to electrical heterogeneity, which in turn, induces non-uniform contraction, relaxation, and filling with further deterioration of global systolic and diastolic function. Cardiac resynchronization therapy (CRT) has resulted in enhanced quality of life, improved symptoms, and improved survival in patients with refractory heart failure due to systolic dysfunction and mechanical dyssynchrony as defined by wide QRS [60, 61]. Although there is a large amount of data reported in several papers, there is still a lot of discussion about this optional therapeutic strategy for patients with HF. The reasons are different: - the percentage of non-responders (34% in the MIRACLE Trial, [62]), whereas there is not a uniform definition of “responder” or “not responder”; - the lack of standardized echocardiographic dyssynchrony parameters for the patient selection either for technical or theoretical limitations (myocardial viability within the paced area, underlying myocardial conditions such as fibrosis and hypertrophy, and location of the pacing lead) [63].

With an eye to the fact that SVR attempts to exclude the scarred tissue, our group reported data from a prospective study conducted on thirty patients undergoing SVR at the Cardiothoracic Center of Monaco [56]. Patients were evaluated using a protocol including simultaneous measurements of ventricular volume and pressure to construct pressure/volume and pressure/length loops. Mean QRS duration was normal (100 ± 17 msec). We showed that intraventricular dyssynchrony and nonuniform contraction develop as a consequence of LV remodeling. This was independent of electrical conduction delay because no patient had left-bundle branch block and preoperative mean duration of QRS was <120 ms. We also showed that SVR produces a mechanical intraventricular resynchronization that improves mechanical efficiency and global LV performance through an improved synergic distribution of regional stress during the isovolumic contraction and relaxation phases. Data on the effectiveness of SVR on LV dyssynchrony have been confirmed by other studies [54, 64].

### SVR and Arrhythmias

The increased risk for life-threatening arrhythmias after a myocardial infarction is related to the complex sequence of events leading to LV remodeling. These events include LV dilatation, which, in turns, increases wall stress and stretch (stretch is arrhythmogenic per sè), and to the presence of electrically unexcitable scars. Our group has previously demonstrated that patients with ventricular arrhythmias (spontaneous or inducible) have end-diastolic and end-systolic volumes significantly larger than those seen in non-inducible patients [65, 66]. SVR has the potential to reduce ventricular arrhythmias by excluding the scar, reducing volumes and thus intraventricular stress and stretch. Ventricular arrhythmias are further reduced by relieving ischemia through complete revascularization and improving mechanical resynchronization. So far, different groups have reported a marked reduction of inducible arrhythmias and a very low incidence of sudden death after SVR [20, 65-67]. This may limit the need for ICD implantation in patients who undergo SVR. The only “negative” contribution, which in fact is not absolutely negative in the “ICD era”, has been reported by O’Neill and co-workers from the Cleveland Clinic. Their report showed that after SVR for ischemic cardiomyopathy, either akinetic or dyskinetic, patients may remain at risk for malignant ventricular arrhythmias and may benefit from prophylactic ICD implantation [68]. Those authors reported a high residual incidence of inducible VT after surgery (42%) and they implanted an ICD in almost 50% of cases in their series. They asserted that ICD implantation may have saved 15 lives because there were 15 appropriate ICD firings. However, the Madit II Trial concluded that an appropriate firing of an ICD identifies patients at increased risk for subsequent heart failure and non sudden cardiac death [69]. Several limitations, including the question-able validity of an EP study as a risk stratifier (different protocol of programmed ventricular stimulation, contraindication to be performed in patients with intraventricular thrombi, left main disease and/or extremely severe LV dysfunction) and the use of different techniques, support the need for caution in interpreting those results.

### SVR and Myocyte Stress

Brain natriuretic peptide (BNP) and its precursor, N-terminal pro-brain natriuretic peptide (NT-pro-BNP), are synthesized in the myocytes and released during hemodynamic stress. This stress occurs when ventricles are dilated, hypertrophic, or subject to increased wall tension [70]. The prohormone is then cleaved by a circulating endoprotease. BNP causes arterial vasodilation, diuresis, and natriuresis, and reduces the activities of the renin-angiotensin–aldosterone system and the sympathetic nervous system in an effort to balance the physiologic abnormalities in heart failure [15].

Previous studies have demonstrated that BNP is a valuable biomarker for LV remodeling as its activation is more pronounced in patients with functional MR rather that in those with organic MR, and it may predict mortality and morbidity in patients with chronic HF [71-74]. Using this relationship, changes in BNP and NT-pro-BNP levels over time could be used to monitor surgical strategies aimed at reversing LV remodeling. The first report addressing this topic was published by Schenk and colleagues from the Cleveland Clinic Foundation [75]. Those authors showed a decrease in plasma levels of BNP by 46% from baseline (before SVR) to 3 months postoperatively. More recently, Sartipy and colleagues reported a similar trend in changes of B-type natriuretic peptides after SVR [76]. Specifically, NT-pro-BNP was significantly reduced by 37% and 51% 6 months after surgery and at late follow-up, respectively (p = 0.03). A similar reduction was observed for BNP levels at the same time-points (by 20% and 34%, respectively), although this reduction was not statistically significant. NYHA improvement correlated significantly with a reduction in BNP and NT-pro-BNP levels 6 months after SVR (r = 0.61 and r = 0.58, respectively), with an increase in ejection fraction (r = - 0.58 and r = - 0.51, respectively) and with a decreased LV end-systolic volume (r = 0.65 and r = 0.62, respectively). Larger studies and longer follow-ups are required to investigate if reduced levels of natriuretic peptides after SVR are associated with better long-term survival.

## CRITICISMS AND FUTURE EXPECTATIONS

To date, the data reported in the literature support the benefit of SVR in improving cardiac function and survival in patients with dilated ischemic cardiomyopathy and heart failure. However, the limitations of different studies are still considerable and make the results presently inconclusive. First, the majority of studies are retrospective and are not powered to address specific end-points either for the number of patients, the limited number of events, or for the length of the follow-up. Second, physiologic studies based on invasive measurements (pressure-volume relationships) have been conducted only on small series of patients. Also, the postoperative measurements were made immediately after the completion of the surgical procedure, or at most, ten days after surgery. Additional information will be provided from these studies performed at later time points. Third, the lack of a control group is one of the major limitations of these studies, and the combination of SVR and coronary artery bypass surgery in the majority of the study groups does not allow for evaluation of the specific role of each procedure. Fourth, different surgical techniques and operative protocol must be taken in account in interpreting the results.

The STICH Trial (Surgical Treatment for Ischemic Heart Failure), sponsored by the National Institutes of Health, is a multicenter, international, randomized trial designed to assess the potential superiority of CABG over intensive medical therapy in improving long-term survival (“the revascularization hypothesis”). It also assesses the benefit of surgical ventricular restoration combined with CABG in improving survival free of hospitalization for cardiac cause compared to CABG alone in patients with LV dysfunction ( EF < 35%) and coronary artery disease suitable for surgical revascularization (“the reconstruction hypothesis”). Patients will undergo cardiac magnetic resonance imaging, echocardiography, neurohormonal and genetic profile, and radionuclide studies to ensure consistent testing practices and standardization of data collection. Testing will also identify eligible patients and address specific questions related to the primary hypotheses. Until the results of this study are available, a close collaboration between cardio-logists and cardiac surgeons in patient selection, perioperative management, and long-term follow-up is critical to definitively change the clinical decision making in the treatment of ischemic heart failure.

## Figures and Tables

**Fig. (1):**
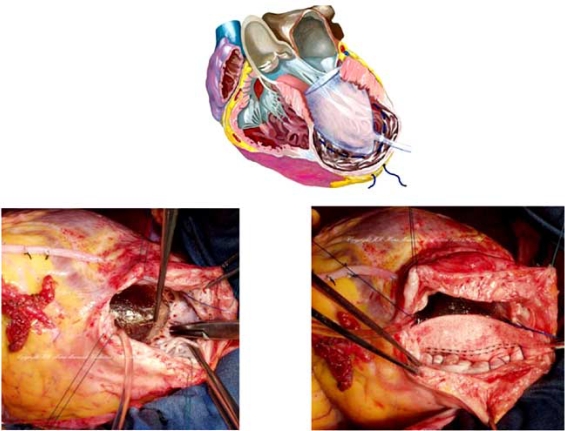
Upper panel - The mannequin positioned inside the ventricle (Schematic); Lower panels - An image of the mannequin from the operating room (*left*); The circular suture follows the curvature of the mannequin to re-shape the ventricle in an elliptical manner. The patch is used to close the ventricular opening (*right*).

**Fig. (2):**
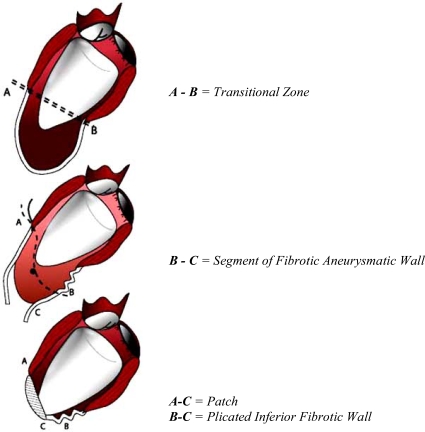
The plication at the inferolateral portion of the ventricle is useful to lift up the new apex.

**Fig. (3):**
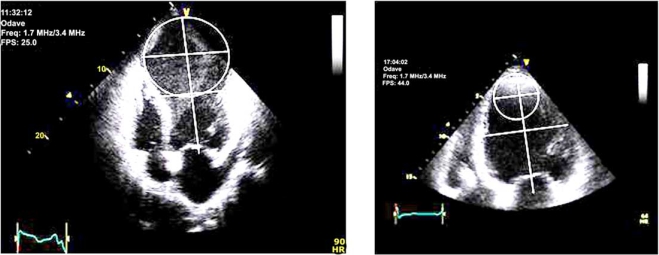
An example of classic aneurysm showing an apical axis that is bigger than the short axis (systolic CI=1.15) (*left*). An example of ischemic dilated cardiomyopathy showing an apical axis that is smaller than the short axis (systolic CI= 0.72) (*right*).

**Table 1:** Summary of Data from the Literature

Author (Ref)	Number of Patients	30-Day Mortality%	EF Change (Absolute Points)	% Survival				
				1 Year	2 Years	3 Years	5 Years	10 Years
Sartipy *et al.* [31]	101	7.9	27±9 to 33±7 (+6)	88%		79%	65%	
Maxey *et al.* [32]	95 (56 with SVR compared to 39 CABG alone)	0	22±11 to 32±9 (+10)		95%			
Mickleborough *et al.* [20]	285	2.8	Not reported	95%			82%	62%
Athanasuleas *et al.* [28]	1198	5.3	29±11 to39±12 (+10)				69%	
Dor *et al.* [33]	870	7.3	Not reported			na		
Di Donato/Dor *et al.* [34]	207	8.1	35±13 to48±12 (+13)	98%			82%	
Cirillo* et al.* [35]	69	4.3	32±4 to 44±7 (+12)		92%			
Menicanti *et al.* [30]	1161	4.7	33±9 to 40±10 (+7)					62%
Ribeiro* et al.* [36]	137	2.6	34±6 to 44±5 (+10)		95%			
Yamaguchi* et al.* [37]	48 CABG(†) vs. CABG +SVR(‡)	?	21±6 to 28±7 (†) 24±7 to 42±9 (‡) (+18)				53% (†) 90% (‡)	
O’Neill *et al.* [29]	220	1.0	21±7 to 25±9 (+4)	92%		90%	80%	
Conte *et al.* [38]	78	7.7	23±9 to 29±10 (+6) *pts in NYHA IV			68%		
Hernandez *et al.* [39] STS Registry	731	9.4	na			na		
